# Determination of serum anti-Mullerian hormone levels in a low-prognosis women treated in-vitro fertilization/intracytoplasmic sperm injection: A cohort study

**DOI:** 10.18502/ijrm.v21i3.13201

**Published:** 2023-04-14

**Authors:** Ferdy Royland Marpaung, Amang Surya Priyanto, Fitri Ayu Kusumawati, Sidarti Soehita, Aryati

**Affiliations:** ^1^Department of Clinical Pathology, Faculty of Medicine, Dr Soetomo Academic Hospital, Universitas Airlangga, Surabaya, Indonesia.; ^2^IVF Morula Clinic, National Hospital Surabaya, Indonesia.

**Keywords:** Prognosis, Assisted reproductive technics, Gonadal hormone, Fertilization in vitro, Reproductive health.

## Abstract

**Background:**

Outcome prediction of participants treated with in-vitro fertilization or intracytoplasmic sperm injection (IVF/ICSI) using anti-Mullerian hormone (AMH) concentration has been widely used. According to the patient-oriented strategies encompassing individualized oocyte number (POSEIDON) definition, low prognosis Bologna responders have changed from poor. This definition divides low prognosis into 4 groups.

**Objective:**

The purpose of this study was to assess blood AMH levels in the group of women treated with IVF/ICSI who were thought to have a low prognosis.

**Materials and Methods:**

A retrospective cohort study among 252 suspected low-prognosis group participants was assessed between January 2016 and December 2019 at Morula IVF, National hospital, Surabaya, Indonesia. Observed AMH serum levels and pregnancy rates were compared among 4 subgroups.

**Results:**

The AMH cutoff value was 1.7 ng/mL with a sensitivity of 86.7% and a specificity of 70% for diagnosing low-prognosis women using POSEIDON criteria. There was no difference in the pregnancy rate between those groups (p > 0.05).

**Conclusion:**

AMH levels may indicate a poor prognosis for women having IVF/ICSI in accordance with POSEIDON guidelines. To predict the poor prognosis in women, the cutoff value must be identified.

## 1. Introduction

Numerous predictive factors can affect whether assisted reproductive technology is successful. This factor has been well studied. Sperm and oocyte quality are the main determinants of success (1). Oocyte quality is determined by a woman's age and ovarian reserve (2, 3). It has been established that parameters such as age, antral follicle count (AFC), follicle stimulating hormone (FSH), day 3 of the inhibin cycle, and random anti-Mullerian hormone (AMH) serum during the menstrual cycle are linked to follicle pool depletion degree (4).

AMH is produced primarily by pre-antral and late-antral follicles. It is one of the most widely used indicators to forecast the success of assisted reproductive technology in terms of pregnancy and cancellation rate in intracytoplasmic sperm injection (ICSI), especially for women who respond poorly to the procedure. However, the use of AMH as a single marker is still controversial. Some studies argued about AMH as a single marker, while another study found variation in the cutoff, sensitivity, and specificity (5, 6).

Women treated with IVF/ICSI were classified as poor responders and normoresponders in 2011 by the European Society of Human Reproduction and Embryology. This term is well known as the “Bologna Criteria.” These criteria help the clinician predict the outcome and give the best counseling to the participants. Age, prior ovarian response, and an ovarian reserve test are among the Bologna criteria for assessing the status of a woman responder treated with IVF/ICSI (7).

The patient-oriented strategies encompassing individualized oocyte number (POSEIDON) criteria have been established. Its criterion redefines “poor responder” to be a “low prognosis” based on quantitative and qualitative data on age and number of aneuploidy embryos, ovarian markers, history of ovarian response to previous therapy and the ability to take oocytes needed to obtain at least one blastocyst euploidy in each patient (8). Therefore, the purpose of this study is to assess the AMH value in women who are thought to have a low prognosis for success with IVF/ICSI therapy using POSEIDON criteria.

## 2. Materials and Methods

### Study design

This study was a retrospective cohort study (historical and prospectively). The sample for this study were participants who came to Morula IVF, National hospital Surabaya, Indonesia, who met the inclusion criteria of females < 45 yr, performed an ultrasound to determine the AFC, and had data on about AMH. Short protocol/GnRH antagonist was given to the participant; cetrorelix 0.25 mg (Cetrotide, Serono International SA, Geneva, Switzerland) was administered once daily (in the morning) when a leading follicle reached a diameter of 12-14 mm; and ovarian stimulation was carried out using rFSH (Gonal F, Serono International SA, Geneva, Switzerland) beginning on cycle days 2-3 at a dose of 225 UI/daily. According to the ovarian response to the medication, the dose was modified for the subsequent days (9). The participants were excluded if diagnosed with polycystic ovarian syndrome (PCOS) or FSH > 20 IU/L.

### Participant

Serum AMH levels were analyzed from January 2016-December 2019 data. Around, 591 individuals received IVF/CSI therapy. A total of 252 participants were suspected of having low prognosis after receiving IVF/ICSI therapy. Participants with poor prognosis were divided into 4 subgroups according to the POSEIDON criteria, namely:

1. Group I: Age < 35 yr, AFC ≥ 5, AMH ≥ 1.2 ng/mL, oocyte numbers in previous cycles < 9

2. Group II: Age ≥ 35 yr, AFC ≥ 5, AMH ≥ 1.2 ng/mL, total oocyte numbers in the previous cycle

3. Group III: Age < 35 yr, AFC < 5, AMH < 1.2 ng/mL

4. Group IV: Age ≥ 35 yr, AFC < 5, AMH < 1.2 ng/mL

**Figure 1:**
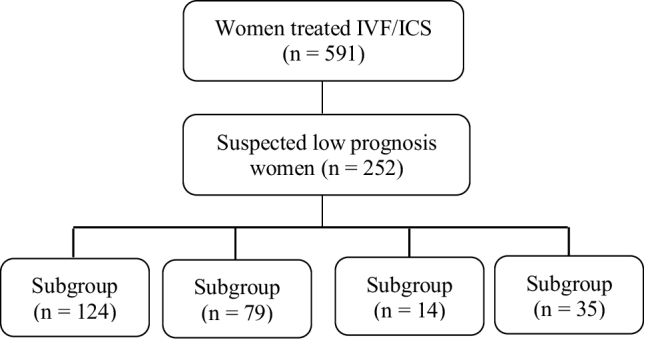
Methodological flowchart of the study.

Examination of serum AMH levels was performed randomly using Vidas (Biomeruex Ⓡ). The AMH examination method is enzyme-linked fluorescent assay. Participants data obtained from medical records, including age, infertility factors, number of IVF/ICSI cycles, body mass index (BMI), AMH, FSH, estrogen (E2), progesterone (P4), and AFC baseline data with 2 ${}^{\text{nd}}$ to 4 ${}^{\text{th}}$ day transvaginal ultrasound by one operator. The outcomes assessed were post-treatment AFC, oocyte uptake, oocyte quality, pregnancy, and number of cycles (Figure 1).

### Ethical considerations

The National hospital Surabaya Ethics Committee in Indonesia granted approval for this study under No. 003/MIS/DIR/2020. Participants received guarantees that their personal data would be kept private and that the research findings would be released under pseudonyms. The participants were also asked to sign consent forms.

### Statistical analysis 

The statistical package for the social sciences (SPSS), SPSS Inc., Chicago, Illinois, USA, version 21.0, was used to analyze the data. To compare the means across more than 2 groups, Kruskal-Wallis was employed. The percentage of pregnancies in groups was compared using chi-square. AMH levels were predicted using the area under the curve (AUC), and the ideal cut-off value was found. It was deemed significant if the p-value was less than 0.05.

## 3. Results

This study comprised 252 participants in total. Demographic data of participants based on 4 sub-groups of suspected low prognosis are shown in table I, figure 1. There were significant differences in the duration of infertility, FSH, oocyte count, and post-treatment AFC (p < 0.05). However, data for age, BMI, luteinizing hormone (LH), and P4 were not significantly different (Table I). Subgroups II and IV had longer infertility than the other subgroups. FSH levels in subgroups II and IV were higher than in other subgroups. The number of oocytes and AFC after subgroups I and II therapy were higher than the other subgroups.

**Figure 2:**
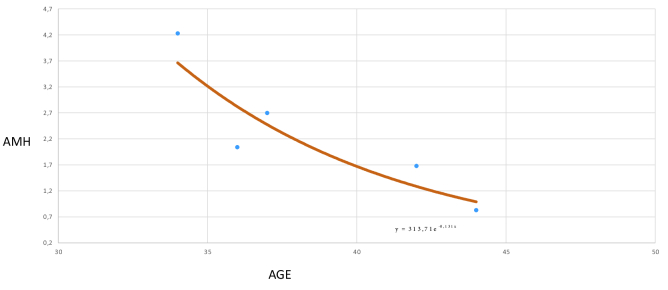
Non-linear graph AMH level and age.

We divided the AMH level into 3 subgroups based on the pattern of prior research subgrouping (6). Of 252 participants, there were 31 participants with AMH levels ≤ 0.39, 93 participants with AMH levels between 0.4-2.1, and 128 participants with AMH levels > 2.1. The parameters of age, duration of infertility, FSH, oocyte count, posttreatment AFC, BMI, LH, P4, and E2 were compared in each group based on the data of AMH level. Data showed that age, duration of infertility, FSH, oocyte count, posttreatment AFC, BMI, and LH were significantly different in each subgroup, while P4 and E2 were not significantly different (Table II). A relationship between age and AMH levels is depicted in figure 2. It is evident that the logarithmic AMH level declines with increasing age (y = 313, 71e^-0comma131x^).

**Figure 3:**
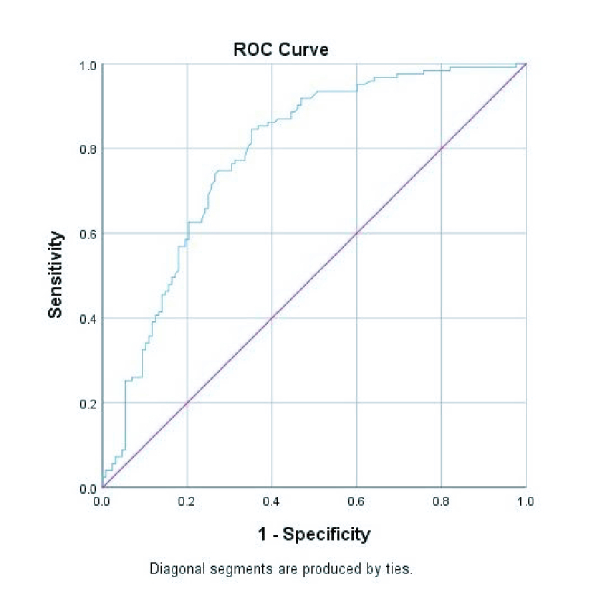
Receiver operating characteristic (ROC) curves of AMH levels in low prognosis.

According to the POSEIDON criteria, the optimal cutoff value for determining women with a low prognosis was 1.7 ng/mL with a sensitivity of 86.7% and a specificity of 70% with an AUC of 0.887 (Figure 3). Interestingly, there were no variations in pregnancy rates across the 4 subgroups (p > 0.05).

**Table 1:** Comparison of parameters for infertility duration, FSH, oocyte count, posttreatment AFC, BMI, LH, P4, and E2 in each subgroup


	**Groups**				
**Parameters**	**I (n = 124)**	**II (n = 79)**	**III (n = 14)**	**IV (n = 35)**	**P-value**
	**BMI (kg/m^2^)**	37.1 ± 5.4	39.0 ± 7.1	40.4 ± 7.6		36.9 ± 5.4	0.07 ${}^{\text{a}}$
**Age (mean)**	30.6 ± 3.0	37.2 ± 2.6	33.3 ± 8.9	38.2 ± 3.8	-
**Infertility period (yr)**	4.4 ± 2.5	8.1 ± 4.5	5.6 ± 2.8	8.9 ± 5.1	< 0.001 ${}^{\text{a}}$
**FSH (U/L)**	5.4 ± 1.7	6.6 ± 2.4	8.1 ± 3.7	9.8 ± 3.9	< 0.001 ${}^{\text{a}}$
**Oocyte count (n)**	14.0 ± 6.6	10.2 ± 7.0	7.0 ± 5.2	0.39 ± 0.35	< 0.001 ${}^{\text{a}}$
**AFC post therapy (n)**	15.5 ± 7.65	12.0 ± 7.9	7.9 ± 6.6	12.3 ± 8.3	< 0.001 ${}^{\text{a}}$
**LH**	3.3 ± 1.3	3.1 ± 1.4	2.5 ± 0.8	3.2 ± 1.3	0.06 ${}^{\text{a}}$
**P4**	0.57 ± 0.37	0.60 ± 0.50	0.50 ± 0.15	0.59 ± 0.56	0.07 ${}^{\text{a}}$
**E2**	34.4 ± 14.2	33.7 ± 16.1	33.9 ± 15.1	34.5 ± 15.7	0.65 ${}^{\text{a}}$
**Pregnant (n)**	19	12	1	1	0.21 ${}^{\text{b}}$
${}^{\text{a}}$ Data presented as Mean ± SD. Kruskal-Wallis. ${}^{\text{b}}$ Chi-square. BMI: Body mass index, FSH: Follicle stimulating hormone, AFC: Antral follicle count, LH: Luteinizing hormone, P4: Progesterone, E2: Estrogen					

**Table 2:** Data parameters for infertility duration, FSH, oocyte count, posttreatment AFC, BMI, LH, P4, and E2 in each AMH subgroup


**Parameters**	**AMH ≤ 0.39 (n = 31)**	**AMH 0.4-2.1 (n = 93)**	**AMH > 2.1 (n = 128)**	**P-value**
**Age (yr)**	40.6 ± 4.96	36.5 ± 4.91	32.0 ± 4.43	< 0.001
**BMI (kg/m^2^)**	36.3 ± 6.6	38.9 ± 6.7	37.4 ± 5.7	0.04
**Time of infertility (yr)**	7.38 ± 5.48	7.71 ± 5.14	5.54 ± 3.77	0.04
**FSH (U/L)**	10.7 ± 1.7	7.3 ± 2.8	5.4 ± 1.7	< 0.001
**Oocyte count (n)**	2.5 ± 2.0	6.9 ± 4.0	15.1 ± 6.8	< 0.001
**AFC post therapy (n)**	3.1 ± 1.5	6.1 ± 2.9	14.6 ± 8.3	< 0.001
**LH**	3.5 ± 1.3	2.9 ± 1.3	3.3 ± 1.3	0.03
**P4**	0.48 ± 0.21	0.62 ± 0.55	0.55 ± 0.36	0.52
**E2**	37.4 ± 17.3	36.5 ± 17.9	32.7 ± 13.6	0.30
Data presented as Mean ± SD. Kruskal-Wallis, AMH: Anti-Mullerian hormone, BMI: Body mass index, FSH: Follicle stimulating hormone, AFC: Antral follicle count, LH: Luteinizing hormone, P4: Progesterone, E2: Estrogen				

## 4. Discussion

This study found an interesting thing, differences in infertility duration in each subgroup. Groups II and IV naturally became a group with long-standing infertility. This may be caused by age in groups II and IV above 35 yr. Previous studies found infertility duration varied in women with a wide range. In posttreatment, the number of oocytes taken was higher in women in subgroups I and II than subgroups III and IV. Indicating age did affect oocyte numbers. This was in accordance with previous research, which found that the decrease in the number of ovarian reserves and oocyte number occurs gradually in accordance with increasing age (10-12). The hormones E2, P4, and LH in these women did not differ in each subgroup. This explains that those hormones have a limited role in ovarian reserve. This finding was in accordance with previous studies, which concluded that E2 and P4 have variations in accordance with the menstrual cycle (6, 13). This study also found a logarithmic decrease in AMH levels with increasing age. Racoubian and colleague found age-specific changes in AMH levels in females aged 17-54 yr modelling. They concluded an indication of AMH levels as an independent indicator of ovarian reserve (14).

The ideal AMH level for a woman with a poor prognosis, according to this study, was 1.7 ng/mL (AUC0.876), with a sensitivity and specificity of 86.7% and 70%, respectively. This result was in accordance with several other studies, which show variations in participants suspected of having a low prognosis. Other studies have found cutoffs to vary from 0.5-2.1 ng/mL (13-16). Since it was introduced in 2011 by the European Society of Human Reproduction and Embryology (ESHRE), “poor responder” participants according to Bologna criteria have been widely studied in terms of the success rate of IVF/ICSI therapy and the failure rate in the form of IVF/ICSI cycle cancellation rates (7, 15, 17). AMH levels were discovered to be crucial in assessing the status of female participants who had IVF/ICSI, both in serum and follicles (16, 18, 19). Many studies have shown that it was important to determine the status of female participants who planned to be treated with IVF/ICSI because it could provide better information for participants about the likelihood of success of IVF/ICSI therapy. Another reason was that patient counseling could perform well based on available data, including AFC, previous history, and AMH levels (6, 16, 18, 19).

Recently, the POSEIDON criteria were adopted, and the Bologna criterion's “poor responder" was changed to “low prognosis". The changing goal remains the same, namely, to provide predictive value to participants. During the IVF/ICSI therapy process, the patient can obtain precise information about their condition and predictions of the success of therapy. POSEIDON criteria add oocyte counts to previous cycles. AMH levels were determined with a cutoff of 1.2 ng/mL (8). However, many studies found that 1.2 ng/mL should not be an “absolute” cutoff value because AMH levels vary depending on ethnic and other factors. The AMH cutoff levels should be determined by the laboratory itself (20-26).

The frequencies of pregnancies in each group did not differ substantially (p > 0.05). This study found the same result as in another study (27). However, prior studies have demonstrated that women with adequate ovarian reserve ( > 5 AFC) have a higher rate of pregnancy and live babies (28). Similar to the results of this study, subgroups I and II had higher AFC than subgroups III and IV, although not significantly different. A higher amount of AFC will result in a higher oocyte yield after increasing the gonadotropin dose, which translates to higher pregnancy and lives birth rate in subsequent cycles (4, 28, 29).

Determination of the optimal cutoff will help the clinician determine predictions for the success of IVF/ICSI therapy. We recommend that every laboratory that performs IVF/ICSI therapy determine their own optimal AMH levels on their examinations and continue to evaluate pregnancy rates, number of cycles, and cancellation of cycles and births in participants who will undergo IVF/ICSI therapy. Proper counseling will help participants understand the conditions and predictions that are best for their success.

## 5. Conclusion

Women having IVF/ICSI may have poor prognoses based on serum AMH levels. Finding the cutoff value is crucial in order to forecast women's poor prognoses. The low-prognosis groups' pregnancy rates were the same. Further evaluation is needed on the cancellation rate of cycles and births in each subgroup based on AMH predetermined.

##  Conflict of Interest

The authors declare that there is no conflict of interest.
